# Reciprocal relations between cardiovascular disease, employment, financial insecurity, and post cardiac event recovery among Māori men: a case series

**DOI:** 10.1186/s13256-023-04202-7

**Published:** 2023-11-12

**Authors:** Samantha Lisipeki, Bridgette Masters-Awatere, Darrin Hodgetts, Tze Vun Liew

**Affiliations:** 1 School of Psychology, Massey University, Albany, New Zealand; 2 School of Psychology, University of Waikato, Hamilton, New Zealand; 3 Te Whatu Ora Waikato District, Hamilton, New Zealand

**Keywords:** Cardiovascular disease, Health disparities, Income, Māori

## Abstract

**Background:**

Disparities in cardiovascular outcomes between Māori and non-Māori persist despite technological advances in the treatment of cardiovascular disease and improved service provision. Little is known about how social determinants of health, such as income [in]security affect Māori men’s access, treatment, and recovery from cardiovascular disease. This paper explores the contexts within which cardiovascular disease is experienced and healthcare becomes embedded.

**Methods:**

This study utilized a case-comparative narrative approach to document and make sense of the patient experiences of four male Māori patients who, in the previous 6 months, had come through cardiac investigation and treatment at Waikato Hospital, a large tertiary cardiac center in New Zealand. Participant accounts were elicited using a culturally patterned narrative approach to case development, informed by Kaupapa Māori Research practices. It involved three repeat 1–3-hour interviews recorded with participants (12 interviews); the first interviews took place 5–16 weeks after surgery/discharge.

**Results:**

Each of the four case studies firstly details a serious cardiac event(s) before describing the varying levels of financial worry they experienced. Major financial disruptions to their lives were at the forefront of the concerns of those facing financial insecurity—as opposed to their medical problems. Financial hardship within the context of an unresponsive welfare system impacted the access to care and access to funding contributed to psychological distress for several participants. Economic security and reciprocal relationships between employers and employees facilitated positive treatment experiences and recovery.

**Conclusion:**

Findings suggest that although multiple factors influence participant experiences and treatment outcomes, financial [in]security, and personal income is a key determinant. The heterogeneity in participant narratives suggests that although general inequities in health may exist for Māori as a population group, these inequities do not appear to be uniform. We postulate diverse mechanisms, by which financial insecurity may adversely affect outcomes from treatment and demonstrate financial security as a significant determinant in allowing patients to respond to and recover from cardiovascular disease more effectively.

## Introduction

The technology for cardiac intervention and surgery has now reached a ceiling in terms of treatment outcomes [1]. Until the development of new advances in physical treatments, further improvements in outcomes for many patients are likely to come from addressing the social determinants of health. For Māori, these social determinants came with colonial processes that confiscated resources and positioned the majority in the lower socioeconomic sectors of society, in risky and low-paid occupations, and in substandard housing. The hospital-based health system in Aotearoa New Zealand (NZ) was also modeled on the British health system. The core orientation remains towards Western notions of treatment [2], with recent extensions into the importance of cultural factors for increasing equity in treatment access and outcomes [3, 4]. A social determinants of health focus is particularly important because many of the factors that shape the health of different population groups reside outside the health system and these factors need to be front of mind in efforts to address differential outcomes for patient populations. Of particular note are Māori males who experience higher rates of coronary heart disease (CHD), case mortality, and have the worst cardiovascular treatment outcomes of all population groups [5]. These trends have persisted despite efforts within the health system to improve the in-hospital provision of cardiac services and to deliver equitable and culturally informed care across patient groups [5, 6].

To understand what is happening for this patient group, health professionals need to look beyond the hospital walls and to consider the contexts within which cardiovascular disease (CVD) is experienced and healthcare becomes embedded. For Māori, these contexts have been established through processes of colonization and corresponding adversities [7], which perpetuate various negative health outcomes [8]. CVD is one of the leading causes of death in NZ men (the other is cancer) being responsible for almost 40% of total deaths in NZ [9]. Māori in all age groups have significantly higher mortality and hospitalization rates for CVD than non-Māori [10]. This article explores Māori men’s experiences of CVD and treatment drawing on a case-based approach to illness accounts that emphasize the importance of lived context [11]. Previous narrative research suggests that CVD disrupts different patient groups in diverse ways [12]. However, little is known about how social determinants of health such as income [in]security affect Māori men’s access, treatment, and recovery from CVD. The analysis presented in this article demonstrates how the work and financial concerns of participating men significantly affects their experience of CVD.

## Method

This study utilized a narrative approach to document and make sense of the narratives of male Māori patients who, in the previous 6 months, had come through cardiac investigation and treatment at Waikato Hospital, a large tertiary cardiac center in NZ. A small number of participants were selected randomly from the Waikato Hospital cardiac rehab database and were engaged in highly in-depth open interviews to gain a more profound understanding of interactions of social determinants of health in patients’ lives. At the first interaction, information sheets were given to prospective participants, informed consent was gained and further appointments for face-to-face interviews arranged. Participant accounts were elicited using a culturally patterned narrative approach to case-based research, which was informed by Kaupapa Māori Research (KMR) practices that involved spending considerable time listening to participants and adherence to Māori cultural principles [13].

Through colonization, the conditions upon which Māori culture was valued, and considered relevant and useful were significantly altered [14] to the point where generations later there are few native speakers of the Māori language. This has resulted in a situation where, for many Māori, navigating a settler society institution such as a hospital can be a disorientating experience which is populated with various cultural obstacles to effective care. With this context in mind, all interviews were conducted in English.

The research is based on three repeat 1–3 hour narrative interviews recorded with four Māori men (12 interviews). Interviews were conducted at patients’ homes kanohi ki te kanohi (face-to-face) and encompassed manaaki ki te tangata (taking care of people) through the sharing of food in each interaction. The interviews were undertaken by S.L. To ensure robust analysis, interviews were audio recorded and transcribed. The narrative analysis was based on an understanding of people as storied beings who make sense of and communicate their experiences by forming personal accounts. We sought to make sense of participant accounts through the use of standard coding techniques that foregrounded key elements or story features, including core characters (doctors, nurses, and family) and contextual considerations (resources, money, and transport), which emerged from how participants make sense of their CVD-related experiences. Thematic categories generated and populated by extracts from the transcripts in the initial analysis by S.L. and D.H were then discussed as a broader research team (S.L., B.M-A., D.H., T.L.) comprising experienced Kaupapa Māori researchers and clinicians.

The following section discusses the results in the form of cases and presents a summary of their narratives. Patients’ names have been anonymized. This procedure received Massey University ethics approval (notification number 4000019378) and was subsequently approved by Waikato District Health Board Quality and Safety Committee (RD018056).

## Results

We present four case studies that illustrate the effects of social determinants of health on patients’ cardiac treatment and recovery. All four patients were working at the time of their CVD event and/or treatment. Henry and Noah continued to receive a work-related income during their CVD treatment and recovery, with Henry owning a trucking business and Noah employed as a factory worker with a supportive employer. In contrast, both Jim and Akul did not have such stability of income and had to rely on accessing the welfare system to cover basic living costs (that is, rent, food, bills, and transport). This is a very stressful system to navigate. Akul worked for a government organization who offered standard sick leave protocols. Jim, a truck driver, was dismissed when he was deemed unfit to drive trucks. Further details of each patient are discussed in the cases.

### Case 1: Jim and his “playing up” heart

Jim (56-year-old Māori male) was initially admitted with cardiogenic shock and severe biventricular impairment in the context of an acute episode of atrial flutter. He had a prolonged hospital stay of 4 weeks, during which coronary angiography demonstrated severe three vessel coronary artery disease (CAD). He was medically optimized with heart failure and secondary prevention medication and following recovery of left ventricular ejection fraction to 50% on echocardiography over 1 year, underwent coronary artery bypass surgery (CABG). He continues to suffer with recurrent paroxysmal atrial fibrillation. The initial interview was undertaken 16-weeks post-CABG.

Jim and Ruby (Jim’s wife) recounted the events surrounding the initial event, which came on suddenly when he was driving a large logging truck through a remote rural area. The remote location shaped Jim’s initial response, rendering the situation particularly dangerous:

*Jim: “I knew there was something wrong, wasn’t sore or anything… I just had to sit there a bit longer… None of the boys was on the radio and couldn’t get a hold of dispatch so just carry on.”*

The cardiac event occurred at work and Jim was forced to carry on driving his fully loaded logging truck at some risk to himself and other road users. His initial response was thus not just determined by his assessment of bodily symptoms. The isolated rural location and nature of his employment shaped his initial response and subsequent access to healthcare [15].

During Jim’s subsequent 1-month stay in Waikato hospital, he was deemed unfit to work. Although Jim had suffered a serious medical event, his narrative emphasizes that the worry and concern he experienced as an inpatient were directed at the major financial disruption to their lives as opposed to his medical problem:

Jim: “The finances have dropped quite badly… I’ve lost my truck license and everything. The boss paid me for so long and that was it. Only while I was in hospital. They [employer] were hoping I would get out and go straight back to work… They’re still hoping I would hurry up with this and go back to work… I was concerned about bills...”

Particularly notable is the stress Jim recounts, which is associated with delayed recovery [16]. Adding to the financial stress is Jim’s need to repeatedly access specialized cardiac care for further follow-up and investigations, located in an urban center more than a 150-km distance from his home [17]. Jim and Ruby discuss how accessing care is particularly difficult from rural areas and drawing on both personal and social resources [18] is crucial.

Jim: “I do all that, work it out whether we got enough money to get up there [hospital] and back… Or if we have to stay up there, I just make the phone call… And they [accommodation organizers^1^] set it up for me… They normally ask you how long, how many days and you just tell them.”

Ruby: “Lucky he’s a good saver… If it wasn’t for him, we probably wouldn’t have gotten anywhere. It’s been hard.”

This excerpt emphasizes the importance of links between accessing care and access to the necessary funds for travel and accommodation [19]. Jim’s quote emphasizes that easily accessible travel grants play an important role for patients accessing care from rural locations, especially for those unable to work due to their cardiac condition. During his interviews, Jim also emphasized a core plot line in his illness narrative centered on income insecurity and how his financial situation changed with CVD and resulted in him“taking a backward step in life” and finding it difficult to “make ends meet.” This financial hardship was emphasized despite his being able to access social welfare support and having private health insurance (*cf*. 20), which did not cover CVD. Engaging with welfare and insurance institutions did not alleviate his stress and rather exacerbated the stress of CVD. As a result of financial insecurity, Jim reports having to engage in paid employment against medical advice:

“All the insurance was a waste of time, me paying all the insurance… So that was another big stress on me... I can’t live on what they give me, what social welfare gives me… That’s why even though I’m not meant to be at work… Couple of days I’ll go to work… Just to make a couple of dollars…”

Engaging in employment is warranted in Jim’s narrative as a necessary means for reducing the level of disruption CVD brought to his life. Jim’s illness narrative aligns with previous research that reports patients with a chronic illness can experience economic hardship despite accessing welfare and financial assistance programs [20]. This case illustrates the difficulty and stress of accessing care for rural patients facing financial insecurity.

### Case 2: Akul and his “repeat heart attacks”

Akul (60-year-old Māori male) first presented with a non-ST elevation myocardial infarction (NSTEMI) in early 2017, which was treated conservatively following angiography. He then suffered a further NSTEMI in August 2017 and had stent implantation to the circumflex artery. He then had further angina, which was diagnosed by a cardiac rehab nurse and following a subsequent positive exercise tolerance test, underwent further stenting to a *de novo* lesion in the right coronary artery (RCA) in January 2018. He was interviewed 8 weeks following his RCA stent.

Akul’s illness narrative focused on a cyclic process of diagnosis, medical intervention, and recovery that he endured three times. When CVD first entered Akul’s conscious life, it invoked a major disruption that lead to him experiencing a prolonged state of uncertainty and increased financial insecurity [21]. The repeated disruptions adversely affected his employment and have left him in a state of limbo. Akul’s account demonstrates how heart disease can force patients to reevaluate how they earn a living [22].

In making sense of the initial cardiac event and his subsequent journey through diagnosis and treatment, Akul makes repeated references to the practical financial consequences of him subsequently suffering multiple cardiac events and undergoing treatment. He recounts being confronted with the predicament of not being well enough to work, running out of sick leave, and struggling to find the money to pay his bills. As a result, he had to navigate a notoriously unresponsive welfare system [23], which then categorized him as being available for work, despite his medical diagnosis saying otherwise:

“After the first stent was fitted it was another 2 weeks off work… But I just didn’t feel right… I’d run out of sick leave at work… I’ve got automatic payments going out of my accounts so I had to go down to social welfare and ask them for some assistance… The only benefit they could give me was “start-up employer” or something… It effectively meant that I was available for job interviews and work. So, [my employer] had me off work because I was too ill, but social welfare had me ready and available for employment…”

Akul experienced numerous setbacks associated with the identification of further heart issues requiring further intervention. Each setback delayed his return to work and ability to move forward with his life. Akul expresses a sense of frustrated eagerness to return to work and the normality in life that he associates with employment:

“We’ve progressed to where they’re finally gonna do the physical test on the treadmill… I’m looking forward to it because if I pass this test okay, the doctor will give me a clearance [to work]. So, I do the test and that’s when they realise that I’m not even close to being well… I didn’t do too well mentally when I went in for the second time.”

Akul’s account emphasizes the psychological impact of suffering setbacks in his return to work and regain some financial stability. Research suggests that anxiety and depression such as that woven into Akul’s narrative are strongly and independently associated with poorer outcomes from CVD [16], including risk of further cardiac events and mortality [24].

As a result of the recurrent presentations, Akul now experiences himself as being trapped in a state of “liminality” that is characterized by uncertainty [21]. He feels lost and reports being increasingly stressed about how he can make a living:

“I just don’t know if I can make a living anymore so, that’s the situation I’m in… Having to choose leaving work not because I want to go, but because I can’t really cope with it... It’s just so damn stressful… Yeah, so for the future as far as working career goes, I don’t know.”

Akul presents his CVD as a disruption to his identity as a productive worker. CVD undermines his financial situation, results in increased life stress, which increase the likelihood of him experiencing further complications [16]. His CVD has not only had immediate effects on his ability to work and support himself financially, but has also led him to live with considerably more stress, uncertainty, and apprehension about the future.

### Case 3: Henry and being “all good”

Henry (65-year-old Māori male) suffered mild exertional chest discomfort over several months prior to presentation. He was admitted with an anterior ST-elevation myocardial infarction (STEMI) and underwent primary percutaneous coronary intervention (PCI) with stent implantation to the left anterior descending (LAD) artery. He was initially interviewed 5 weeks post discharge.

Henry is the first of two participants whose experiences of CVD were more positive in comparison to Akul and Jim as he experienced less employment and financial disruption. The gradual onset of manageable symptoms, the swift medical intervention and his short stay in hospital have left him feeling less stressed than the previous two participants. The limited illness disruption that Henry experienced is associated with his owning a trucking business and having employees who looked after work in his absence.

Henry recounted the period leading up to his heart event where he slowly began to experience chest pains over several months. Because he was still able to function normally and had responsibilities for running his company, he did not initially seek care, attributing symptoms to more mundane health concerns:

“I was getting sorta chest pains few and far between… I just thought it was just heart burn… Nothing major…”

Henry recounted how CVD had not brought a lot of additional stress or worry into his life. Henry did not articulate worries about employment in the same way as Jim and Akul:

“I wasn’t worried about work. I knew that work would carry on, the workers would be there if I couldn’t make it, and which they’re still doing it now so I didn’t worry about that…”

Henry presents as a person who has experienced a temporary setback and who is on track to return to his normal routine. In the absence of employment and financial concerns, he is able to quickly restory himself as someone who thought he was healthy, but who was impaired briefly by illness. While acknowledging the importance of work for generating an income for himself, Henry also emphasized his role as a good employer who was more concerned about how his ‘brief’ illness might disrupt the incomes and lives of his employees:

“I got six drivers there that are depending on the work that I give them… It’s just part of life I suppose. You gotta work to survive isn’t it really? They come first; my workers come first… I suppose it’s their livelihood…”

In this extract Henry presents an account of his CVD that features a concern for the livelihood of his employees rather than himself. In doing so, Henry invokes a reciprocal relationship with his employees who afford him the luxury of a continued income and the time to recover from his heart attack.

### Case 4 “For what it was…it was a positive experience”

Noah (53 year Māori male) initially presented with likely Staphylococcus aureus endocarditis and moderate to severe mitral regurgitation in 2011, following an episode of lumbar osteomyelitis. He was initially treated medically and was monitored with serial yearly echocardiograms. He developed worsening mitral regurgitation and left ventricular enlargement and in 2017 underwent elective cardiac catheterization for work-up to surgery. This confirmed severe mitral regurgitation and single vessel coronary artery disease in the RCA and underwent elective mitral valve replacement and CABG in 2018. The first interview, was performed 12-weeks post discharge from surgery.

In contrast to the other three cases, Noah’s cardiac condition was diagnosed and monitored for several years until his condition worsened. Despite this lengthy period before surgical intervention, like Henry, Noah’s illness narrative is one of minimal disruption, due to a supportive workplace and continued financial security. He works in a dairy factory driving forklifts. Noah recounts moving through treatment and recovery with a sense of ease and limited stress and anxiety.

While managing his heart condition and awaiting further treatment, Noah’s symptoms appeared gradually and approximately 3 years following the initial detection of his heart condition:

“After a few years it just got worse and worse and just getting breathless at times. But mostly at work going up the stairs and stuff…”

Noah recounts his deteriorating condition through the realm of employment and notes the impact in this context in terms of breathlessness and tiredness impacting his performance. In discussing the processes of having his heart condition monitored over a considerable period of time, Noah reported few concerns in relation to accessing care or continuing with his life. Instead, facilitating factors, including a flexible employer, enabled Noah to attend cardiac appointments with minimal hassle:

Noah: “If I had an appointment yeah, I’d just make sure I’d be there and keep everything rolling along how it was supposed to be I suppose…If it was during work time, work would just let me take a couple of hours off or the rest of the day off. And so yeah, it wasn’t too hard.”

Noah’s narrative demonstrates that his supportive employment situation made his condition both psychologically and structurally less disruptive. Compared with Jim and Akul, Noah has not had to suffer the socioeconomic consequences of illness. He was able to be absent from work and undergo treatment and recovery while remaining on full pay. Noah also discussed how the “special sick leave” offered by his employer facilitated his positive treatment experience and recovery:

“Work gave me a bit of time off, so it’s been really good not having to rush back… Didn’t have to worry about the money sorta thing… That was really good cos it was peace of mind that, ya know. All the bills were gonna be paid and there’s gonna be food on the table... It’s really a load off your mind... Just concentrate on getting better. Cos you don’t wanna be worrying about, ya know, when the next loaf of bread is coming from or whatever...”

Noah went on to reflect openly on the benefits of his situation and how things might have been different if he had financial concerns and needed to rush back to work. Noah’s employment buffers him from some of the negative impacts of CVD by affording him a sense of financial stability and continuity in life. His financial stability allows him to fully participate in follow-up and rehabilitation programs.

## Discussion

Combined, these four cases illustrate how CVD affects patients’ lives, poses contrasting levels of disruption, and are responded to in both heterogeneous and similar ways. These cases demonstrate a diverse experience of CVD among Māori men and show that Māori patients are not a homogeneous group [13]. Nevertheless a key determining feature linking positive and negative experiences was employment and financial [in]security. These were not issues that this research set out to investigate, but emerged in different ways through participants’ narratives. Financial security assists Henry and Noah in withstanding the disruption of initial diagnoses, treatment and recovery. They are able to concentrate on self-care and rehabilitation both physically and psychologically. As a result, there is minimized overall disruption to their lives.

In contrast, for Jim, limited employer support contributes to a potentially dangerous delay to access care. For Jim and Akul, the financial hardships caused by illness impair their abilities to care for their physical and psychological needs during illness, treatment, and recovery. It compounds their psychological burden, which manifests through multiple expressions of anxiety and uncertainty. These are serious health concerns as stress and depression are significantly and independently associated with poorer CVD outcomes [24], including major adverse cardiovascular events (MACE). It also drives them to undertake work against medical advice, for which their medical condition renders them physically incapable, a more significant concern for patients employed in manual professions, of which Māori patients are more overrepresented [25, 26]. It forces patients to prioritize financial concerns over health and recovery and makes accessing follow-up and rehabilitation difficult. Comparison of the narratives of those who need financial support with those who do not highlights the critical importance of social welfare provisions and simplicity of access to care and the inadequacy of the current provisions to meet the requirements of both Jim and Akul to manage their recovery in a less stressful and healthier manner.

The heterogeneity in participant narratives suggests that although general inequities in health may exist for Māori as a population group, these inequities do not appear to be uniform. It signals the need for service providers to be more nuanced when considering the needs of Māori men suffering from CVD and recovery, and to not put all men in the same adverse outcome category simply in accord with their ethnicity. Responses need to be tailored to the needs of less affluent cohorts within this population group. However, as Māori are more strongly represented in low/precarious income groups they are more likely to be adversely affected by financial insecurity as a whole [25].

## Conclusion

According to previous research, addressing disparities in cardiovascular outcomes necessitates formulating and delivering models of care in hospitals that better cater to the needs of particular population groups [27, 28]. Adding to earlier research, the present study supports the need for clinicians to place more emphasis on issues such as employment and financial insecurity that exist beyond the realm of the hospital. Given the current state of surgical interventions reaching a ceiling in terms of efficacy [1], to gain further improvement in patient outcomes we need to address the insecurities raised by our participants. Easily accessible means-tested financial support for acute cardiac patients may help reduce stress and depression and improve engagement with follow-up, rehabilitation programs, and healthy lifestyle changes.

This study is the first to explore potential causes of poorer cardiovascular outcomes in Māori using qualitative narrative and Kaupapa Māori methodology. Kaupapa Māori methodology has allowed us to elucidate and explore in depth the principle concerns of the patients. This article presents findings with a small group of men, which needs to be elaborated with further research. We postulate diverse mechanisms by which financial insecurity may adversely affect outcomes from treatment and demonstrates financial security as a significant determinant in allowing patients to respond to and recover from CVD more effectively.

## Data Availability

The datasets generated and/or analyzed during the current study are not publicly available due to confidentiality clauses with the research agency agreements but can be made available, with protections, from the corresponding author on reasonable request.

## Abbreviations

CAD: Coronary artery disease
CABG: Coronary artery bypass surgery
CHD: Coronary heart disease
CVD: Cardiovascular disease
KMR: Kaupapa Māori research
LAD: Left anterior descending
MACE: Major adverse cardiovascular event
NSTEMI: Non-ST elevation myocardial infarction
PCI: Percutaneous coronary intervention
RCA: Right coronary artery
STEMI: ST-elevation myocardial infarction

## Footnotes

1 National Travel Assistance Scheme is funding eligible patients to help with the expenses of travel and accommodation related to seeking specialist care [19].
